# Metabolic deficits and immune dysfunction in aging people living with HIV

**DOI:** 10.1097/IN9.0000000000000071

**Published:** 2025-11-17

**Authors:** Daniela Frasca, Suresh Pallikkuth

**Affiliations:** 1 Department of Microbiology and Immunology, University of Miami Miller School of Medicine, Miami, FL, USA; 2 Sylvester Comprehensive Cancer Center, University of Miami Miller School of Medicine, Miami, FL, USA

**Keywords:** aging, B cells, HIV, metabolism

## Abstract

Cellular metabolism is crucial for energy production, which regulates cell function and survival. In recent years, the importance of metabolism in modulating immune cell proliferation, differentiation, and function has become a prominent area of research. However, little is still known about the metabolic regulation of B cell function and humoral immunity, both in healthy individuals as well as in those with various conditions and diseases. In this viewpoint, we will discuss the current understanding of immunometabolic regulation of humoral responses in aging people living with HIV, and in people without HIV. We propose the possibility to target metabolic molecules and pathways to prevent the negative effects of aging and HIV and progress towards an overall better immune system, not only in individuals with HIV but also in those living with other inflammatory conditions and diseases.

## 1. Background

People living with HIV (PWH) experience systemic chronic inflammation known as “inflammaging” ^[1]^, with accelerated immune aging and chronic immune activation (IA) ^[2]^, which are key drivers of impaired immunity to infections and vaccines, as well as dysfunction across both the innate and adaptive immune cell compartments. Although the use of antiretroviral therapy (ART) reduces IA, residual IA often persists even in virally suppressed PWH. The persistent IA contributes significantly to HIV disease pathogenesis and progression, immune dysfunction, and the development of non-AIDS co-morbidities, including cardiovascular and renal diseases, cognitive impairment, and cancers ^[3]^. In addition, AIDS-related opportunistic infections may further promote chronic inflammation and remain a leading cause of mortality in PWH ^[4]^.

Aging further exacerbates these immune abnormalities. Chronic HIV infection accelerates many of the immune changes typically seen with physiological aging ^[5]^. Studies have shown that ART-treated PWH exhibit increased frequencies of T cells with an immune aging phenotype compared to people living without HIV (PWoH), along with elevated expression of senescence-associated IA markers (CD38^+^HLA^−^DR^+^CD28^−^/CD57^+^) in both CD4^+^ and CD8^+^ T cell subsets ^[6]^, which are strongly associated with increased risk of non-AIDS multimorbidity. The susceptibility of CD4 T cells to HIV infection correlates with a hypermetabolic status ^[7]^. Similarly, our published findings have demonstrated that the circulating B cell pool of PWH, as compared to that of PWoH, is enriched in B cell subsets with characteristics of immunosenescence and IA, as they express transcripts for multiple senescent and inflammatory markers, and we have shown that the expression of these transcripts is associated with the expression of metabolic markers, suggesting that IA in PWH is metabolically supported ^[8]^.

## 2. Immunometabolic support of B cell immune activation and immunosenescence

Aging is associated with several metabolic changes, including decreased insulin sensitivity ^[9,10]^, mitochondrial dysfunction ^[11,12]^, and impaired nutrient uptake ^[13]^. These alterations result in the release of metabolites involved in glucose and fatty acid metabolism into the blood. Although a few studies have identified and characterized metabolic signatures of aging ^[14–16]^, gaps still exist, and it is not clear how the identified metabolites regulate biological aging-related processes, longevity, and survival. Inflammaging is characterized by increased uptake of nutrients and enhanced glycolysis, which provides a rapid but inefficient energy pathway that supports anabolic processes and promotes the secretion of inflammatory mediators, thereby fueling inflammaging ^[17]^. These observations underscore the importance of studying immunometabolism to better understand the metabolic drivers and pathways that trigger immune activation and function. We believe it is critical to assess an individual’s metabolic status to identify pathways that support or impair immune competence. In PWH, metabolic abnormalities have been linked to virus-induced irreversible tissue damage in viremic individuals, ART-induced effects in virally suppressed individuals, gut microbial translocation, chronic co-infection with latent viruses, and gut microbial dysbiosis, all of which may influence the metabolic status and functional capacity of immune cells ^[18]^, as well as to HIV-associated conditions ^[19]^. However, the impact of aging on these metabolic pathways and the functional status of the immune cells in PWH remains poorly understood and is the central focus of our research.

Similar to HIV, infection with cytomegalovirus, severe acute respiratory syndrome-coronavirus-2 (SARS-CoV-2), Zika virus, or Hepatitis B virus also impacts immune cell function through metabolic pathways, and this occurs during both acute and chronic infection ^[20]^.

As to autoimmune diseases, it has been shown that alterations in cell metabolism support dysfunctional innate and adaptive immune cells characterized by the dysregulation associated with autoreactive immune cells, thus sustaining loss of immunological self-tolerance ^[21]^.

We have shown that HIV infection induces metabolic reprogramming in B cells, rendering them hyper-inflammatory and hypermetabolic. In particular, B cells isolated from the peripheral blood of young and elderly PWH, as compared with those from PWoH, express higher levels of transcripts encoding pro-inflammatory cytokines (tumor necrosis factor [TNF-α] and interleukin [IL-6]), microRNAs (miR-16 and miR-155), markers of senescence and proliferation arrest (p16^INK4^ and p21^CIP1/WAF1^), and key metabolic enzymes. These include lactate dehydrogenase A (which converts pyruvate into lactate), a marker of anaerobic glycolysis, and *PDHX*, a component of the pyruvate dehydrogenase complex that converts pyruvate into acetyl-CoA, which supports oxidative phosphorylation and mitochondrial fitness PWH ^[8]^. This altered metabolic phenotype contributes to B cell dysfunction in PWH, characterized by being detrimental for B cells and leading to dysfunctional humoral immunity (less protective responses to infections and vaccinations, while pathogenic/autoimmune responses are increased), and we have identified for the first time a relationship between B cell IA and B cell intrinsic metabolism. More recently, we have identified and characterized novel mechanisms through which metabolic dysfunction (increased anaerobic glycolysis and lactate secretion) accelerates and exacerbates defects in immune cells from healthy, uninfected individuals, leading to decreased secretion of protective antibodies to the influenza vaccine ^[22]^.

Among B cell subsets, double negative B cells, a highly pro-inflammatory population, are the major contributors to this hyper-inflammatory and hypermetabolic profile ^[23,24]^. We consider that the hypermetabolic status of B cells from individuals with inflammatory conditions and diseases, including PWH, supports the ongoing persistent IA and compromises their function.

**Figure 1.**
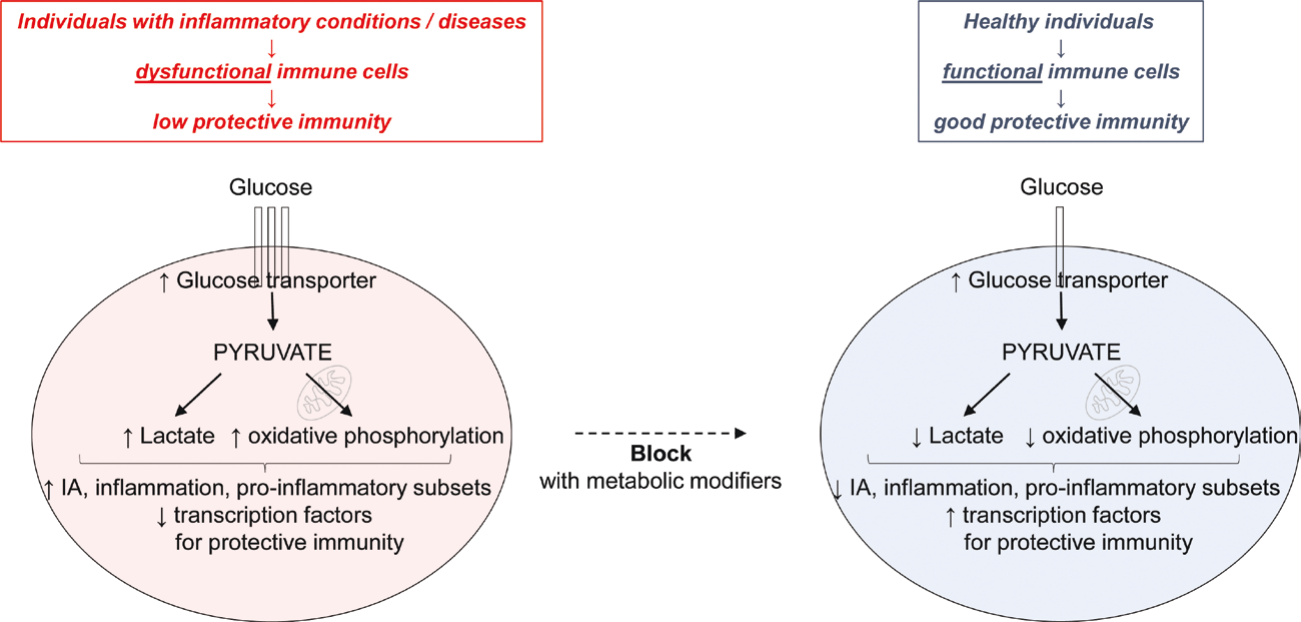
**Immune cells (B/T/monocytes) from individuals with inflammatory conditions and diseases (left), as compared with those from healthy individuals (right), are hypermetabolic, and this phenotype is associated with higher levels of IA, intrinsic inflammation, higher frequencies of pro-inflammatory subsets, and with the secretion of inflammatory (TNF-α/IL-6) and metabolic mediators (lactate**). These immune cells are also low in transcription factors for protective antibody responses. The phenotype and the transcriptional profile of immune cells from inflamed individuals can be blocked, at least in vitro, if immune cells are pretreated with metabolic modifiers. IA, immune activation; IL, interleukin; TNF, tumor necrosis factor. Modified from Frasca et al^[25]^.

Based on our previous findings, we propose the model shown in Figure 1. Briefly, immune cells from individuals with inflammatory conditions (aging, obesity, and type-2 diabetes) and diseases (chronic viral infections and autoimmune diseases) exhibit a hypermetabolic phenotype that supports intrinsic inflammation associated with IA and with the secretion of inflammatory and metabolic mediators, as well as of pathogenic antibodies. Importantly, intrinsic inflammation can be blocked, at least in vitro, by pretreating immune cells from these individuals with the metabolic modifiers metformin, rapamycin, vitamin D, and lactate inhibitors. The inhibition of different metabolic pathways (glycolysis, the tricarboxylic acid cycle, the pentose phosphate pathway, fatty acid oxidation/synthesis, and amino acid metabolism) has already been proposed to be an efficient tool to shape the immune response, and many small molecules are available to specifically target these pathways ^[26]^. Table 1 summarizes the conditions and diseases in which these metabolic modifiers are currently tested for their effects to reduce intrinsic inflammation and IA in immune cells.

**TABLE 1:** Conditions and Diseases in Which Metabolic Modifiers are Employed.

Metabolic Modifier	Condition	Disease
Metformin	Aging Type-2 diabetes	Inflammatory bowel disease Hidradenitis suppurativa Gout COVID-19
Rapamycin	Aging	Systemic lupus erythematosus Amyotrophic lateral sclerosis periodontal disease Chronic fatigue syndrome
Vitamin D		Systemic lupus erythematosus Type-1 diabetes
Lactate inhibitor AZD3965^a^		Solid tumors
Lactate inhibitor AR-C155858^a^		Rheumatoid arthritis
Lactate inhibitor SLC5A12^a^		Rheumatoid arthritis
Lactate inhibitor SLC5A12^a^		Sjögren’s disease
Lactate inhibitor FX11^b^		Solid tumors

a Inhibitors of lactate transporters that catalyze the transport across the cell membrane of many monocarboxylates, including lactate (also known as MCTs, monocarboxylate transporters).

b Inhibitor of lactate dehydrogenase, the enzyme that converts pyruvate into lactate in the cytosol.

## 3. Current and future studies

Our current research investigates how aging affects the serum and gut metabolic profiles of PWH compared with PWoH, and how these metabolic changes impact the phenotype, function, and transcriptional profiles of immune cells involved in protective responses to infections and vaccines. Work from other groups has already identified some metabolites (glycerophospholipids) that increase similarly with age in both PWoH and PWH ^[27]^, while other metabolites like glutamine and *N*-glycans ^[28]^ increase with age only in the blood of PWH, but not in that of healthy controls, suggesting that these metabolites may contribute to accelerated biological aging and increased inflammaging in PWH. Altogether, these studies aim to define a unique metabolic signature for each individual, which may establish a potential link between the systemic metabolic status of the individual and the intrinsic metabolic phenotype and function of immune cells. This approach is particularly novel, as immune cell function is shaped not only by substrate availability but also by metabolite-driven signaling pathways. In addition to identifying metabolic signatures, our research, as well as that from other groups, seeks to uncover cellular, molecular, and metabolic mechanisms of aging in PWH that may inform the development of potential therapeutic strategies. Specifically, we aim to selectively modulate immune cell function using senolytics and/or metabolic modifiers. This will have a dual impact on the reduction of the hypermetabolic and hyper-inflammatory status of immune cells and on the improvement of immune responses, at least in vitro. The possibility that metabolic pathways in immune cells can be therapeutically targeted to mitigate cell-intrinsic IA and restore immune function provides a promising strategy to counteract age-related immune decline in a large number of inflammatory conditions and diseases. Our studies, aligned with National Institute on Aging’s Strategic Directions, are highly relevant for public health, as they seek to better elucidate the biology of aging and to develop interventions to counteract the deleterious consequences of systemic inflammation on both duration and quality of life.

## Author contributions

DF and SP wrote the viewpoint and were involved in funding acquisition.

## Conflict of interest

The authors declare that they have no conflict of interest.

## Funding

This research is funded by the National Institute on Aging (NIA) 1R01AG086071-01A1.
